# Synergistic optical, dielectric and visible-light photocatalytic enhancement in Mo-modified BaTiO_3_ nanostructures

**DOI:** 10.1038/s41598-025-22201-0

**Published:** 2025-10-27

**Authors:** Mohammed Ahmed Wahba, Saad Mabrouk Yakout, A. M. Youssef

**Affiliations:** Inorganic Chemistry Department, National Research Centre (NRC), El Buhouth St., Dokki, Cairo, 12622 Egypt

**Keywords:** Perovskite materials, Multi-functional compositions, BaTiO_3_, Dielectric properties, Visible-light photocatalysts, Dye degradation, Chemistry, Materials science

## Abstract

Molybdenum-doped barium titanate (BaTiO_3_) nanostructures were fabricated by a solid-state reaction and evaluated for simultaneous enhancement of dielectric and optical performance. X-ray diffraction confirmed that progressively higher Mo content drives a tetragonal to cubic phase transformation, evidencing effective lattice tuning. Complementary SEM and EDX analyses showed well-defined grain morphology and uniform elemental distribution, verifying successful Mo incorporation. XPS detected Mo in mixed valence states (Mo^3+^/Mo^4+^/Mo^6+^) together with Ti^3+^ species, implicating abundant oxygen vacancies that promote charge transport and surface reactivity. Dielectric measurements revealed a marked rise in room-temperature permittivity accompanied by lower loss, indicating improved polarization dynamics. UV–vis diffuse-reflectance spectra displayed a red-shifted absorption edge and a band-gap narrowing from 3.24 eV (pristine BaTiO_3_) to 2.92 eV (MBT4), thereby extending visible-light harvesting. All Mo-doped BaTiO_3_ samples exhibited notable visible-light photocatalytic performance, with the 3% Mo-doped sample (MBT3) achieving significantly enhanced degradation of Congo red dye under direct sunlight. Notably, MBT3 demonstrated about 90% degradation efficiency within 60 min, compared to the slower response of undoped BaTiO_3_, and the corresponding photocatalytic rate constant increased from 0.01754 min^−1^ (pure BTO) to 0.03673 min^−1^, underscoring the superior reactivity and light-harvesting capability imparted by Mo incorporation. These results demonstrate that Mo incorporation simultaneously tailors BaTiO_3_ crystal structure, electronic structure, and dielectric response, positioning the material as a promising multifunctional candidate for sustainable energy and environmental applications.

## Introduction

Perovskite-type oxides with the general formula ABO_3_ have emerged as a promising and adaptable family of materials, extensively studied for their potential in photocatalytic, electronic, and energy-related applications^1–4^. Their flexible crystal structure allows for a wide range of elemental substitutions at the A- and B-sites, enabling fine-tuning of their physicochemical properties. Among these, BaTiO_3_ (BTO) stands out as a notable perovskite material due to its ferroelectric nature, high dielectric constant, thermal stability, and wide band gap (~ 3.2 eV), which make it suitable for various multifunctional applications^5,6^. BaTiO_3_ can exist in several polymorphs, including cubic, tetragonal, orthorhombic, and rhombohedral phases, with the tetragonal and cubic forms being the most relevant. Particularly, the tetragonal ferroelectric phase exhibits a bent band configuration, which generates an internal electric field that assists in separating photo-generated charge carriers, thereby reducing recombination and enhancing photocatalytic performance^7,8^.

With the rapid industrialization, increasing volumes of hazardous organic pollutants, especially dyes are being released into water bodies, posing serious environmental and health concerns. Conventional treatment methods are often ineffective or expensive for complete degradation of these persistent contaminants^9–11^. In response, advanced oxidation processes (AOPs), particularly photocatalysis, have gained considerable attention as green, sustainable, and efficient technologies for wastewater treatment^12–16^. Photocatalysis utilizes semiconductor materials under light irradiation to generate reactive oxygen species capable of mineralizing organic pollutants. The key to maximizing this process lies in selecting and engineering a suitable photocatalyst with an optimal band gap, strong visible light absorption, and high charge separation efficiency.

While halide perovskites have demonstrated remarkable optical properties and carrier transport capabilities, their vulnerability to moisture, structural instability, and toxicity concerns pose major limitations to practical deployment^17^. In contrast, oxide perovskites like BaTiO_3_ offer a more robust, non-toxic alternative with superior dielectric and structural stability, making them ideal for multifunctional applications^18^. However, BaTiO_3_ has a wide band gap limits its ability to harness visible light, but this can be addressed by incorporating transition metal dopants that not only reduce the band gap but also enhance the charge transport and separation dynamics. A variety of dopants such as Li, Cr, Mn, Fe, Co, Cu, Ce La and N have been explored to enhance photocatalytic properties of BaTiO_3_^8,19–24^ Notably, transition metals with multiple oxidation states and ionic radii compatible with the B-site (Ti^4+^), can introduce defect levels or intermediate states in the band structure, improve visible-light response, and suppress electron–hole recombination. Their 3d and 4d orbitals can interact with the Ti–O lattice, potentially enhancing both electronic conductivity and optical absorption. At the same time, the dielectric properties of BaTiO_3_, such as its relative permittivity (ε_r_), dielectric loss (tan δ), and polarization response, can be greatly influenced by the nature and concentration of dopants^25,26^. Doping introduces localized lattice distortions and defect dipoles, which contribute to enhanced polarization mechanisms and increased dielectric constants, key for high-performance energy storage and ferroelectric applications.

Numerous studies have demonstrated that doping with aliovalent cations significantly alters its functional behavior by introducing lattice strain, modifying electronic band structure, and influencing defect chemistry. For instance, copper doping in BaTiO₃ predominantly substitutes at the Ti sites, where Cu²⁺ ions stabilize the hexagonal phase through Jahn–Teller distortion effects. Compared to other 3d dopants, Mn, Fe or Ni, Cu is more effective in promoting the cubic-to-hexagonal phase transition, though its solubility in the BaTiO₃ lattice is relatively limited^27^. A study on A-site doping in BaTiO₃ revealed that Gd-doped samples exhibit larger c/a ratios and cell volumes compared to La-doped ones. Interestingly, this trend contradicts predictions based on ionic radii and tolerance factor arguments, and the anomalous behavior was attributed to cation-size mismatch at the A-site, which induces local strain and affects the Curie temperature^28^. A study on double-doped BaTiO₃ (Bi and Mn up to 0.5 mol%) demonstrated that increasing Bi content enhanced tetragonal anisotropy by shrinking the a-axis and elongating the c-axis, thereby stabilizing and expanding the ferroelectric tetragonal phase region. Structural and magnetic analyses confirmed that Bi³⁺ substitution at the A-site induced strong off-center displacement due to its 6s² lone-pair electrons, which in turn influenced Mn valence states and lattice fluctuations, distinguishing its effect from conventional Jahn–Teller distortions^29^. Cation–anion co-doping with rare-earth elements, particularly La, has been shown through DFT studies to significantly modify the electronic structure of BaTiO₃, enhancing its optical absorption and photocatalytic activity. Notably, 25% La substitution at the Ti site introduced intermediate states within the bandgap, enabling efficient visible-light absorption and improved water-splitting performance^30^. In another study, Cu²⁺ and Cu²⁺/W⁴⁺ incorporation into BaTiO₃ induced gigantic relative permittivity and enhanced ac electrical conductivity, accompanied by the formation of a secondary α-Ba₂TiO₄ phase (17–25%), which decayed upon heating. Structural and morphological analyses showed distinct particle networks and grain morphologies depending on dopant type, while optical measurements revealed a significant red-shift of the UV absorption edge due to doping and codoping effects^23^. A first-principles study on co-doped BaTiO₃ revealed that V–M″ co-doping, where (M″ is 3d or 4d transition metal), is more effective than Nb–M″ in narrowing the bandgap and enhancing visible-light absorption, as it generates shallow states near the band edges instead of deep levels. The optimal configuration was identified as V–Cr co-doping, which lowers the bandgap by ~ 0.4 eV through synergistic electronic interactions, offering a promising strategy for tailoring BaTiO₃ for visible-light-driven solar applications^31^. Importantly, the effect of doping is strongly dependent on concentration: low levels may only induce slight lattice strain, while higher levels can generate oxygen vacancies, secondary phases, or defect states that drastically influence functional properties. Therefore, systematic exploration of BaTiO₃ with different dopants and doping levels is crucial for understanding structure–property relationships and optimizing performance in electronic, dielectric, and photocatalytic applications.

In this study, BaTiO_3_ was synthesized using a simple one-step solid-state reaction method and doped with varying wt% of Mo ions to systematically evaluate their impact on the structural, morphological, optical, dielectric, and photocatalytic properties. Solid-state route exhibited a fruitful rout to tune structural and optical properties along with stable photocatalytic activity. Unlike widely reported sol–gel or hydrothermal methods, the solid-state approach employed here offers a scalable and cost-effective pathway to achieve uniform dopant incorporation. Molybdenum (Mo) as a dopant for BaTiO_3_ has compatible ionic radius and multiple oxidation states, allowing effective substitution at the Ti^4+^ site. This substitution is expected to promote vacancies formation and intermediate energy states, enhancing both visible light absorption and charge carrier separation. As a result, Mo doping with its 4d-orbitals not only is expected to improve the photocatalytic activity but also to enhance the dielectric and permittivity properties of BaTiO_3_. The Photocatalytic performance was assessed through the degradation of Congo red dye under direct solar irradiation, aiming to identify the most effective dopant in promoting photocatalytic efficiency and contributing to the development of multifunctional perovskite-based materials for environmental and energy applications.

## Experimental

### Synthesis and measurements of pure and mo doped BaTiO_3_ samples

The synthesis of pure and doped barium titanate (BaTiO_3_) was achieved through a conventional solid-state reaction technique, a widely adopted method known for its simplicity, scalability, and ability to produce phase-pure ceramic materials. All reagents used were of high analytical grade to ensure the reliability and reproducibility of the synthesized compositions. Specifically, barium nitrate (Ba(NO_3_)_3_, 99.6% purity) and titanium dioxide (TiO_2_, 99.9% purity) served as the primary precursors for Ba and Ti ions, respectively, in the formation of the undoped BaTiO_3_ structure. For the preparation of Mo-doped compositions, molybdenum pentachloride (MoCl_5_, 99.9%), was chosen as the Mo precursor. The synthesis process began by weighing stoichiometric amounts of Ba(NO_3_)_3_ and TiO_2_ in a 1:1 molar ratio for the pure BaTiO_3_ sample. The powders were manually ground using an agate mortar and pestle for approximately 5 h to ensure thorough mixing and achieve uniform particle distribution. The homogenized mixture was then subjected to a two-step calcination process in a muffle furnace under atmospheric conditions. Initially, the powder was calcined at 500 °C for 2 h to decompose the nitrate and initiate solid-state diffusion. After cooling, the powder was further heated to 1200 °C and maintained for 5 h to complete the reaction and promote the crystallization of the BaTiO_3_ perovskite phase. For the synthesis of Mo-doped BaTiO_3_, the same base procedure was followed with the incorporation of dopant materials. Accurately weighed amounts of MoCl_5_ (corresponding to 1, 3 and 4 mol% of Ti) were added to the Ba(NO_3_)_3_-TiO_2_ mixture prior to the grinding step. The inclusion of dopants at this early stage allowed for uniform distribution throughout the matrix during mechanical mixing. Subsequent calcination steps were identical to those used for the pure sample, ensuring comparable thermal conditions across all samples for fair evaluation. The samples were coded according to the Mo doping percentage as BT, BTM1, BTM3 and BTM4.

### Instruments and characterization

X-ray diffraction (XRD) using a PANalytical X’Pert PRO instrument was employed to confirm phase purity and study materials structure. The microstructure and surface morphology of the resulting powders were examined through scanning electron microscopy (SEM, model Quanta 250 FEG). The UV–visible light absorption spectra were recorded using a JASCO double-beam spectrophotometer (model V-570 UV–Vis–NIR), enabling the determination of the optical band gap energies through the Kubelka–Munk function and corresponding refractive index values using established empirical equations. For dielectric analysis, cylindrical pellets of approximately 1 cm in diameter and 2 mm in thickness were prepared from each composition. These pellets were characterized at room temperature using a precision LCR meter (Hioki 3532-50, Japan) to assess the frequency-dependent dielectric properties.

The photocatalytic performance of BaTiO_3_ and Mo-doped samples was evaluated by monitoring the degradation of Congo red (CR) dye under natural sunlight. For each test, 0.05 g of the photocatalyst powder was dispersed in 100 mL of an aqueous CR solution (10 mg L⁻¹), and the suspension was continuously stirred to maintain uniform exposure. The photocatalytic process was initiated by exposing the solution to sunlight, (12:00 p.m. to 3:00 p.m.) between 11:00 AM and 2:00 PM, when sunlight intensity is relatively stable, at the NRC institute in Cairo (latitude 30° 3′ 45.47″ N, longitude 31° 14′ 58.81″ E) in mid-May. The average solar intensity during irradiation was 660–680 W/m^2^ and aliquots of 5 mL were withdrawn at regular intervals (10, 30, 45 and 60 min). These samples were centrifuged for 10 min to remove catalyst particles, and the remaining dye concentration was determined by measuring the absorbance at the maximum wavelength of CR (497 nm). The photocatalytic efficiency (PE) was calculated using the relation PE = A_t_/A_0_ = C_t_/C_0_, where A_0_ and C_0_ represent the initial absorbance and concentration, and A_t_ and C_t_ represent the values at a given irradiation time, respectively.

## Results and discussion

### X-ray diffraction analysis

**Fig. 1:**
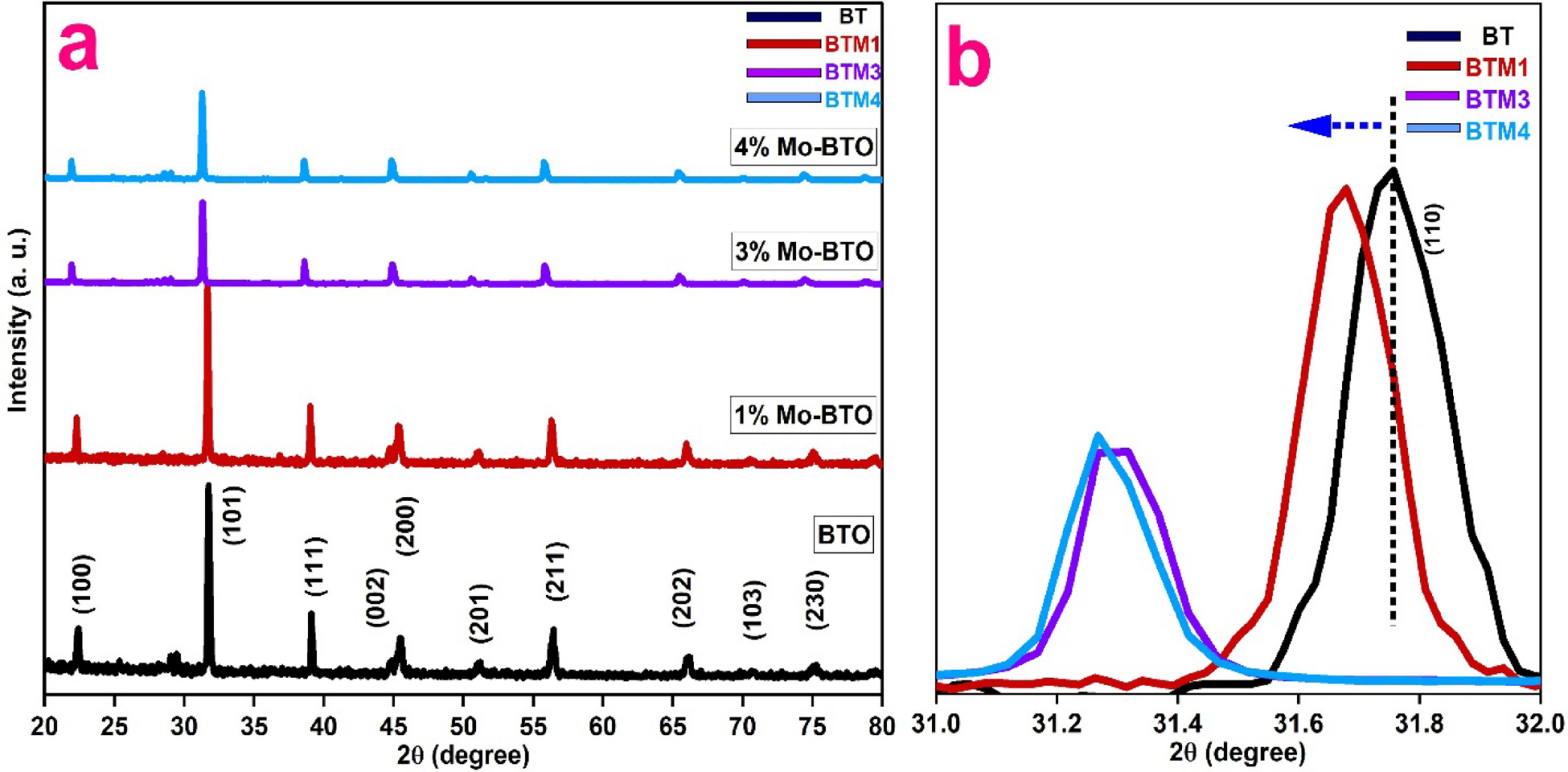
(**a**) X-ray diffraction patterns of the synthesized pure BaTiO_3_ (BTO) and Mo doped BaTiO_3_ at 1, 3 and 4% respectively (BTM1, BTM3 and BTM4) samples; (**b**) Magnified view of the (110) diffraction plane within the 2θ range of 31.0 to 32.0°.

Figure 1a demonstrates the patterns of the X-ray diffraction (XRD) analysis of the pure and Mo doped BaTiO_3_ (BTO) samples inside 2-theta 20–80°. Based on the X-ray diffraction lines of pure BaTiO_3_, the diffraction peaks with 2-theta and equivalent crystallographic planes = 22.36° (100), 31.73° (110), 39.09° (111), 44.76° (002), 45.39° (200), 51.09° (201), 56.352° (211), 66.02° (220) and 75.07° (310) were evident, indicating the formation of tetragonal-phase of BaTiO_3_ (space group P4mm, JCPDS Card file No. 05-0626). In precise, the X-ray diffraction peak nearby 2-theta = 45° was divided into two lines or peaks (Fig. 1a), which confirms that the manufactured pure BaTiO_3_ powder has the tetragonal structure (JCPDS Card file No. 05-0626). The same XRD peaks were detected for 1% Mo doped BaTiO_3_ structure with also observing the splitting around 2-theta = 45° into (002) and (200) crystallographic planes, verifying tetragonal phase structure of this composition. Conversely, the splitting of the peak around 2-theta = 45° was disappear and the (002) and (200) peaks were merged into one diffraction peak with increasing the Mo concentration to 3 and 4 wt%, signifying the formation of the cubic-phase of BaTiO_3_ structure (JCPDS NO. 31–0174).

The tetragonal-to-cubic phase transition observed with increasing Mo content can be ascribed to surpassing a critical doping threshold, beyond which lattice strain and defect formation energetically favour the cubic structure. This threshold exceeds the 2 mol% limit reported in a previous study^8^, where no transition was detected, and is consistent with the absence of such transformation at 1 mol% Mo in the present work. The incorporation of Mo ions in multiple oxidation states (Mo³⁺, Mo^4^⁺, Mo^5+^) generates oxygen vacancies and induces local lattice distortions, collectively driving cubic phase stabilization. XPS analysis further supports this mechanism by confirming enhanced defect chemistry that facilitates structural reorganization. These results reflect that the Mo concentration has a significant effect on the crystal structure of the produced BaTiO_3_ semiconductor.

**Fig. 2:**
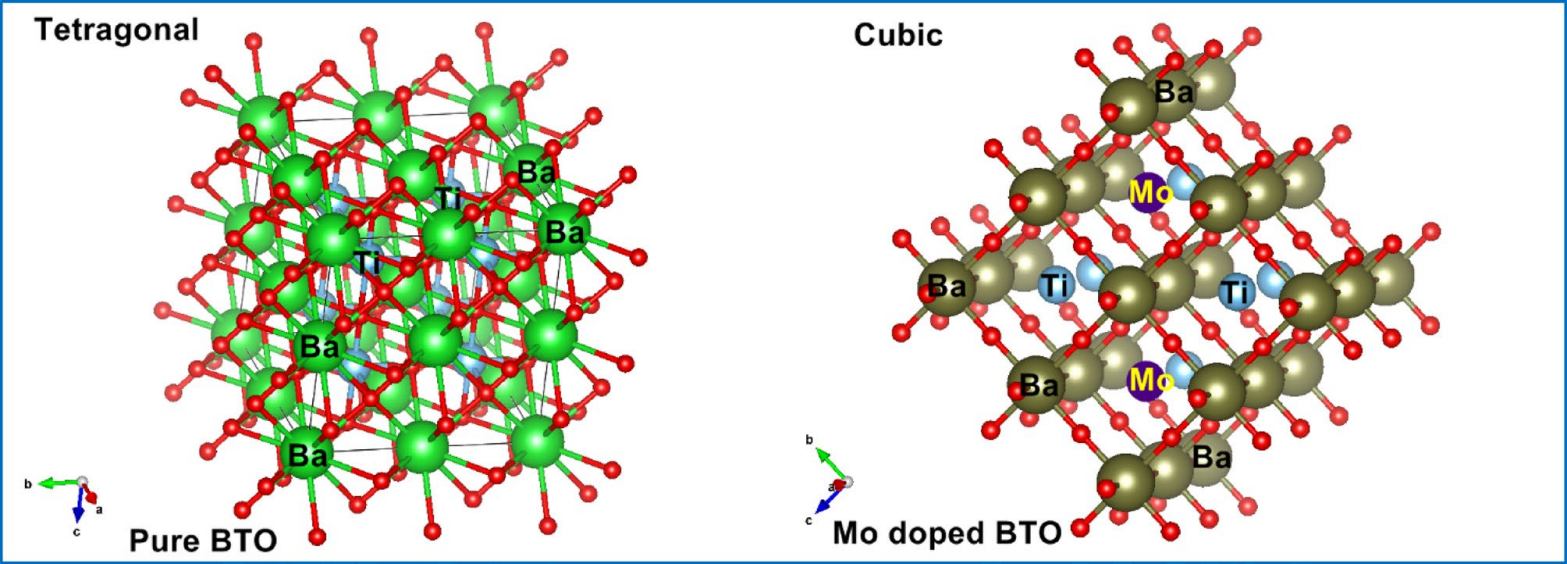
The geometrical crystal structure of tetragonal pure and cubic Mo doped BaTiO_3_ (BTO) samples.

Additionally, as illustrated in Fig. 1b, a magnified view of the (110) diffraction plane within the 2θ range of 31.0° to 32.0° reveals noticeable peak shifts toward lower angles for the Mo-doped BaTiO_3_ samples compared to the undoped counterpart. This shift toward lower 2θ values is indicative of an expansion in the unit cell volume of the BaTiO_3_ lattice. Such expansion is consistent with the substitution of Ti^4+^ ions (ionic radius 0.605 Å, coordination number VI) by Mo^3+^ (0.69 Å, VI) and Mo^4+^ (0.65 Å, VI), whose ionic radii are larger than Ti^4+^ but still significantly smaller than Ba^2+^ (1.61 Å, CN = XII), suggesting that the dopant ions preferentially occupy Ti^4+^ sites. The observed lattice expansion and peak shifts strongly support the incorporation of Mo ions into the Ti^4+^ positions within the crystal structure. Furthermore, this substitution leads to the formation of oxygen vacancies to maintain charge neutrality, as Mo^3+^ and Mo^4+^ possess lower oxidation states than Ti^4+^. These oxygen vacancies act as charge compensating defects arising from the aliovalent doping process^32^. The geometrical crystal structure of pure and Mo doped BaTiO_3_ (BTO) samples were drawn using Diamond software as shown in Fig. 2, to illustrate cations coordination.

The lattice parameters of pure and Mo-doped BaTiO_3_ compositions, calculated using Unit Cell software from XRD patterns, reveal distinct structural transitions influenced by Mo substitution. As shown in Table 1, the pure BaTiO_3_ (BT) sample exhibited a tetragonal crystal structure, characterized by slightly different lattice constants in the c-axis compared to a and b (a = b = 3.9922 Å, c = 4.0401 Å), with a corresponding unit cell volume of 64.3908 Å³. This is consistent with the known room-temperature structure of BaTiO_3_. Upon doping with 1 wt% Mo (BTM1), the structure retained its tetragonal symmetry but showed slight elongation in the a-axis (a = 4.0176 Å), indicating lattice distortion likely due to substitution of Ti^4+^ (ionic radius ~ 0.605 Å) with slightly larger or multivalent Mo ions: Mo^3+^ (0.69 Å, VI), Mo^4+^ (0.65 Å, VI) and Mo^6+^ (0.59 Å, VI). The unit cell volume increased to 65.03 Å³, suggesting lattice expansion from Mo incorporation and possible generation of oxygen vacancies to maintain charge neutrality. Interestingly, for the 3 wt% and 4 wt% Mo-doped samples (BTM3 and BTM4), the structure transitioned to cubic symmetry as confirmed by equal lattice constants in all directions (a = b = c = 4.0299 Å for BTM3 and 4.0410 Å for BTM4). The corresponding unit cell volumes were 65.45 Å³ and 65.99 Å³, respectively. This phase transformation from tetragonal to cubic can be attributed to increased Mo content, which suppress tetragonality. The expansion of the unit cell volume also confirms successful substitution of Mo into the Ti sites and possibly enhanced ionic displacements or vacancy formation. The observed structural evolution highlights the sensitivity of the BaTiO_3_ lattice to cationic substitution, where even a small concentration of Mo ions can significantly influence crystal symmetry, lattice distortion, and unit cell size. These structural changes are closely tied to enhanced dielectric and photocatalytic properties, as discussed in the following sections.

**Table 1:** Crystallite size D (nm), lattice parameters (a, b, c), unit cell volume (V), phase of pure and doped BaTiO_3_ samples.

Samples	D nm	a Å	b Å	c Å	V Å^3^	Phase
BT	55	3.9922	3.9922	4.0401	64.3908	Tetragonal
BTM1	51	4.0176	4.0176	4.0287	65.0282	Tetragonal
BTM3	61	4.0299	4.0299	4.0299	65.4472	Cubic
BTM4	66	4.0410	4.0410	4.0410	65.9891	Cubic

The crystallite sizes of pure and Mo-doped BaTiO_3_ samples were estimated from the X-ray diffraction (XRD) peak broadening using the Debye–Scherrer equation: D = Kλ/βcosθ^33,34^. The calculated crystallite sizes are summarized in Table 1. These results indicate that low Mo doping (BTM1) slightly decreased the crystallite size (51 nm) compared to the pure BT sample (55 nm). However, with increased Mo content (BTM3 and BTM4), the crystallite size increased, reaching 66 nm for 4% Mo. The slight reduction in crystallite size observed for the BTM1 sample compared to pure BaTiO_3_ can be attributed to localized lattice strain introduced by initial Mo-doping (1%). At this low doping level, the strain is not sufficiently relaxed, limiting crystallite growth. In contrast, higher Mo concentrations promote strain relaxation through defect generation, oxygen vacancy formation, and partial grain coalescence, thereby mitigating the size-reducing effect observed in BTM1. This interplay between lattice strain and relaxation mechanisms explains the observed trend in crystallite size across the doped series. This suggests that higher Mo doping not only promotes lattice expansion, as confirmed by unit cell volume increases, but also facilitates crystal growth, possibly by reducing grain boundary energy or enhancing sintering activity during calcination. Moreover, the transformation from tetragonal to cubic phase with Mo doping might provide a more energetically favorable configuration for larger crystallites to form.

### Morphology study

**Fig. 3:**
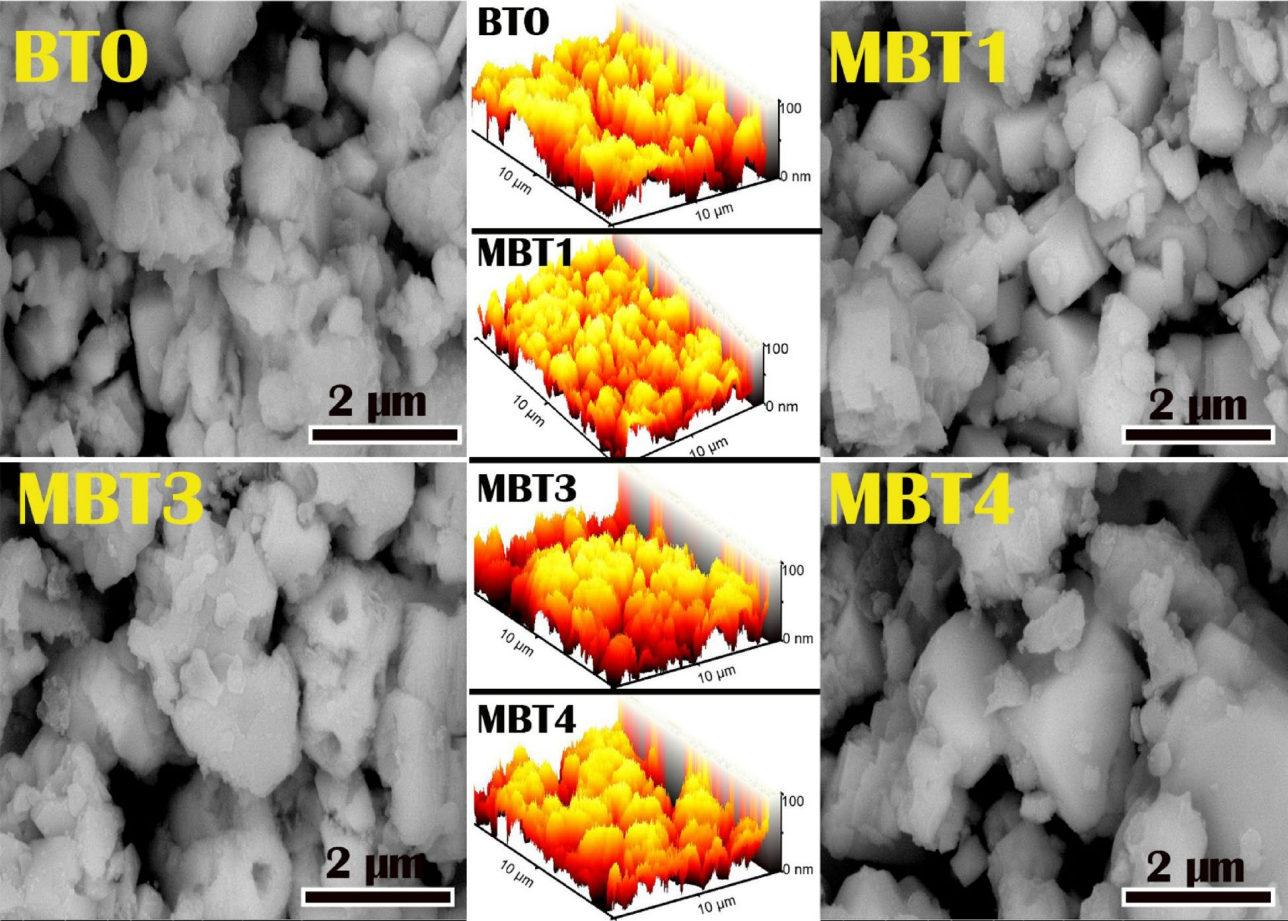
SEM micrographs and 3D images of pure and Mo doped BaTiO_3_ powders.

The SEM images (Fig. 3) provide insight into the morphological evolution of BaTiO_3_ (BT) and Mo-doped BaTiO_3_ samples as a function of increasing Mo content. The undoped BT sample reveals a surface composed of irregular, densely agglomerated particles with indistinct grain boundaries, indicating a less controlled crystallization process and heterogeneous particle growth. Upon doping with 1% Mo, the particles become more defined with improved faceting and reduced agglomeration, suggesting that the introduction of Mo ions contributes to enhanced grain development and better crystallinity. However, as the Mo doping increases further to 3%, the surface morphology begins to deteriorate; particle boundaries become less distinct and the grains more irregular, hinting at the onset of structural disorder possibly due to excess dopant incorporation disrupting the lattice and affecting grain growth dynamics. This trend continues in the 4% Mo-doped sample, where the microstructure becomes increasingly coarse and agglomerated, with several grains appearing to partially coalesce or sinter together. The smooth and rounded features, combined with a loss of uniformity, imply that higher Mo content adversely impacts the crystallization behavior, potentially due to the formation of more lattice defects. Overall, the SEM observations demonstrate that while low levels of Mo doping can enhance particle definition and crystallinity, excessive doping compromises structural uniformity, indicating a delicate balance between dopant level and microstructural integrity.

**Fig. 4:**
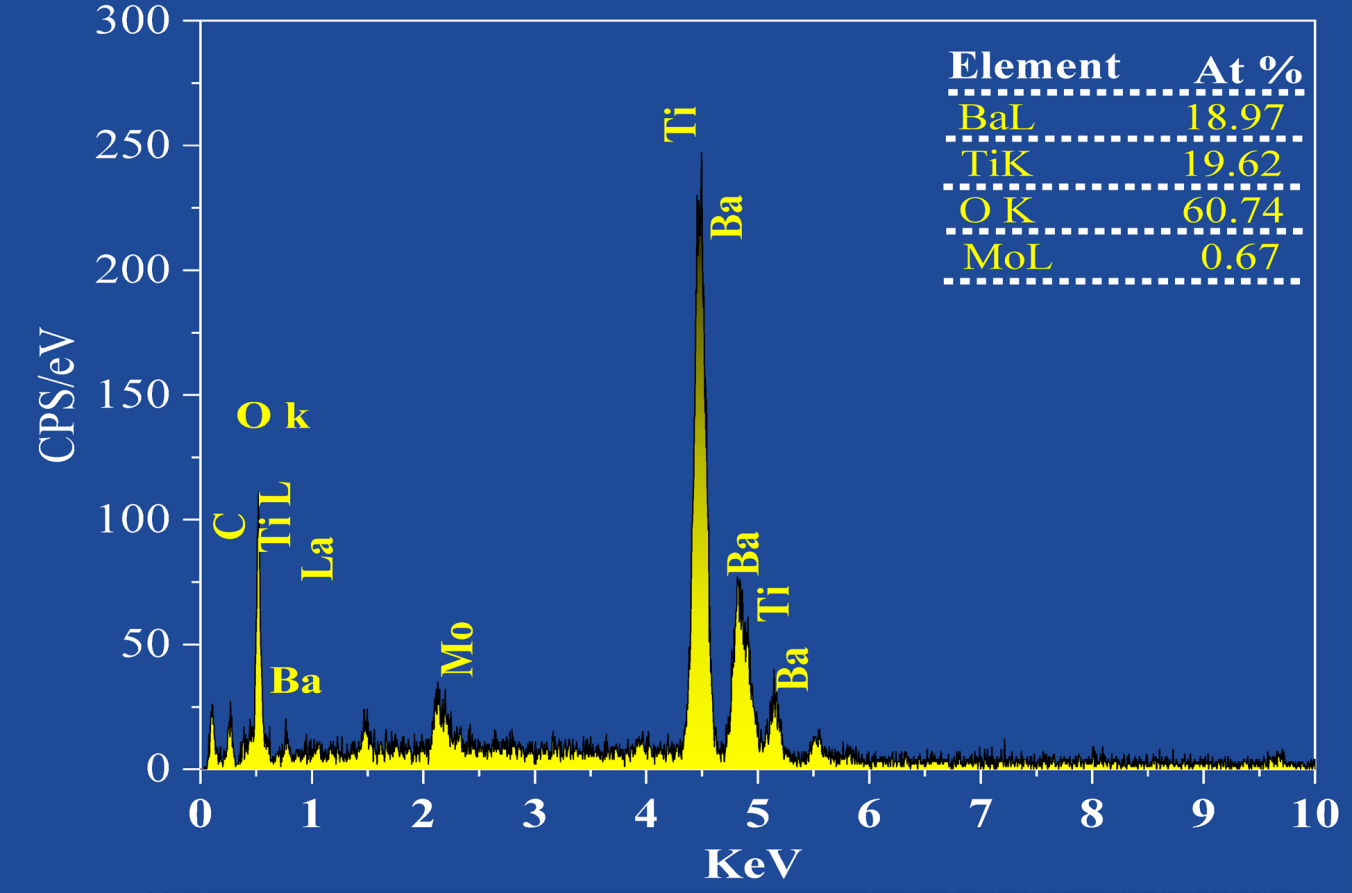
EDX of 1% Mo doped BaTiO_3_ (BTM1) sample.

The EDX spectrum (Fig. 4) confirms the elemental composition of the Mo-doped BaTiO_3_ sample. The major peaks correspond to Ba, Ti, and O, which are the primary elements of the BaTiO_3_ perovskite structure. The strong Ti and Ba peaks indicate a well-formed crystal phase, while the prominent oxygen signal reflects the oxide nature of the material. Importantly, a distinct Mo peak is observed, confirming successful doping. Overall, the EDX analysis verifies the elemental purity and confirms Mo incorporation into the BaTiO_3_ lattice.

### Compositional study

**Fig. 5:**
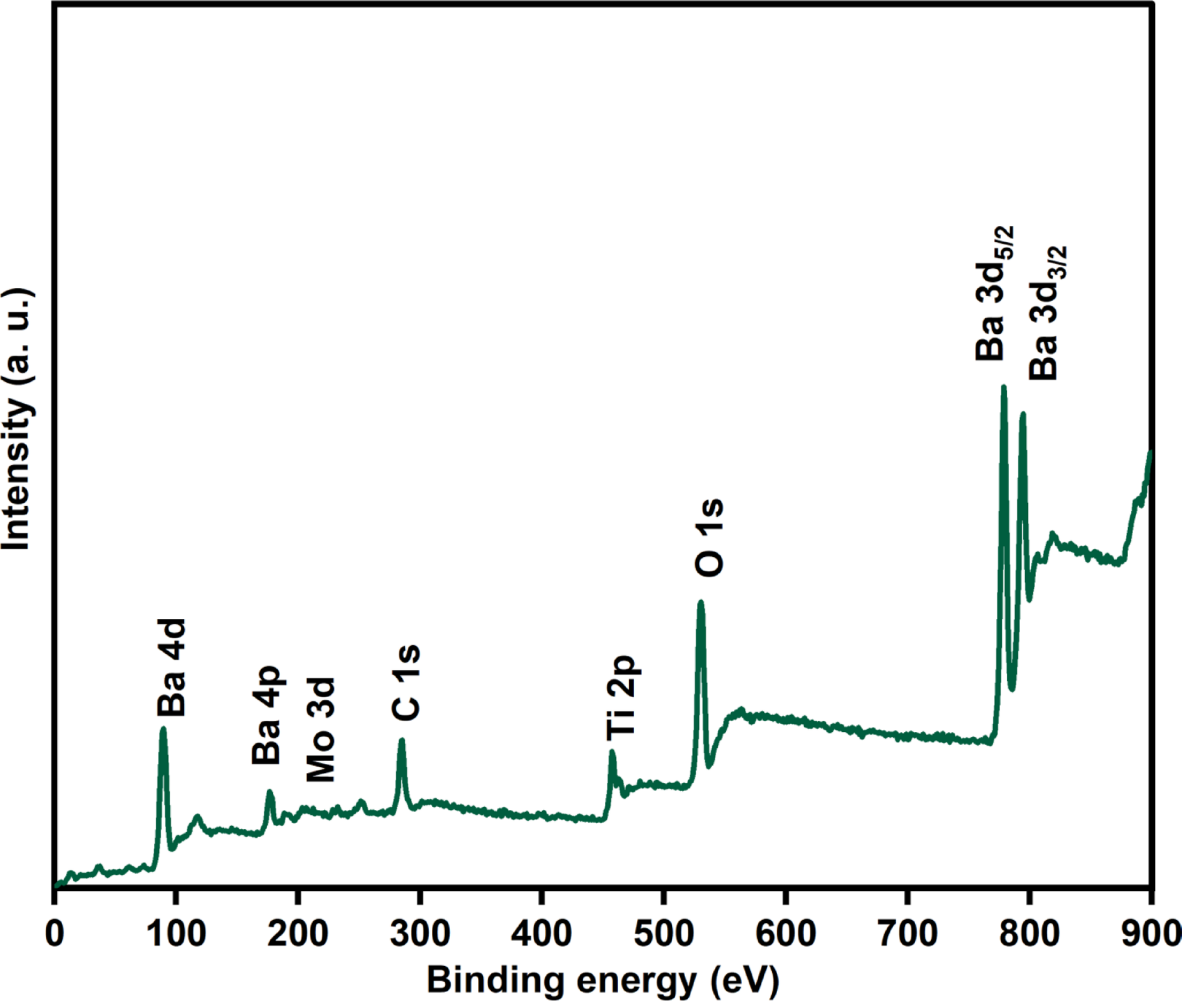
X-ray photoelectron (XPS) survey spectra of BTM3 sample.

Figure 5 displays the survey scan X-ray photoelectron signals of Mo doped BaTiO_3_ composition in band energy range of 0-900 eV. The binding-energy scale was referenced to the adventitious hydrocarbon C 1s component at 284.8 eV. In addition to carbon, Ba, Ti, Mo and O elements were detected without recorded of other impurities lines.

**Fig. 6:**
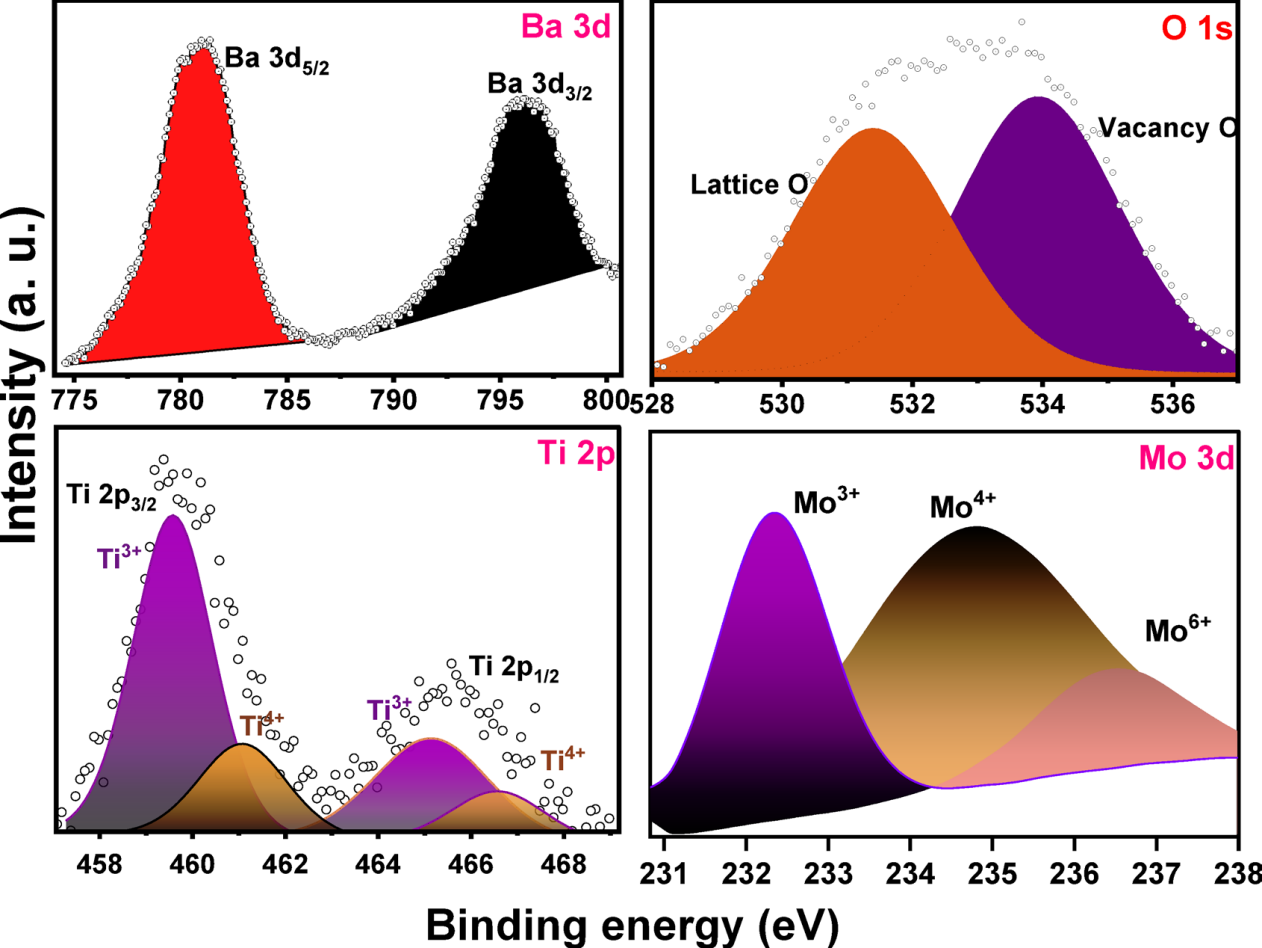
X-ray photoelectron (XPS) spectra of BTM3 sample: (**a**): Ba 3d, (**b**): Ti 2p, (**c**): O 1s states, (**d**): Mo 3d.

Figure 6a–c gives the high-resolution core level XPS spectra of Ba-3d, Ti-2p and O-1s peaks. Figure 6a demonstrates that the 3d signals of barium (Ba) component was split into two binding energy peaks located at 779.77 eV and 795.35 eV, corresponding to 3d_5/2_ and 3d_3/2_, respectively, which is linked with the Ba^2+^ perovskite building^35,36^. The scan spectrum of high resolution XPS of Ti 2p reveals four key peaks from the states Ti 2p_3/2_ and Ti 2p_1/2_ for the Mo doped BaTiO_3_ composition positioned at 457.75 and 461.08 eV related to Ti^3+^ oxidation state while the two binding energy peaks observed at 459.58 eV and 463.54 eV were assigned to Ti^4+^ oxidation state. These binding energy values are in agreement with the previously reported X-ray photoelectron signals of Ti^3+^ and Ti^4+^ states^35,36^. It seems that some of Ti cations with oxidation valence state of + 4 in Mo doped BaTiO_3_ composition were converted to Ti^3+^ oxidation valence state so as to balance the oxygen vacancies existing in this composition. The O1s core level signal as given in (Fig. 6b) was resolved into two binding energy peaks situated at 531.4 and 533.93 eV. The binding energy signal positioned at 531.4 eV was ascribed to the lattice oxygen in Mo doped BaTiO_3_ composition while the binding energy peak at 533.93 eV may be owing to the oxygen vacancies present at the surface of the particles^37^. Figure 6d represents the high resolution of Mo 3d core level splitting which exhibits three binding energy peaks located at 231.8, 233.83 and 236.44 eV which corresponding to Mo^3+^, Mo^4+^ and Mo^6+^, respectively^38^.

### Optical study

**Fig. 7:**
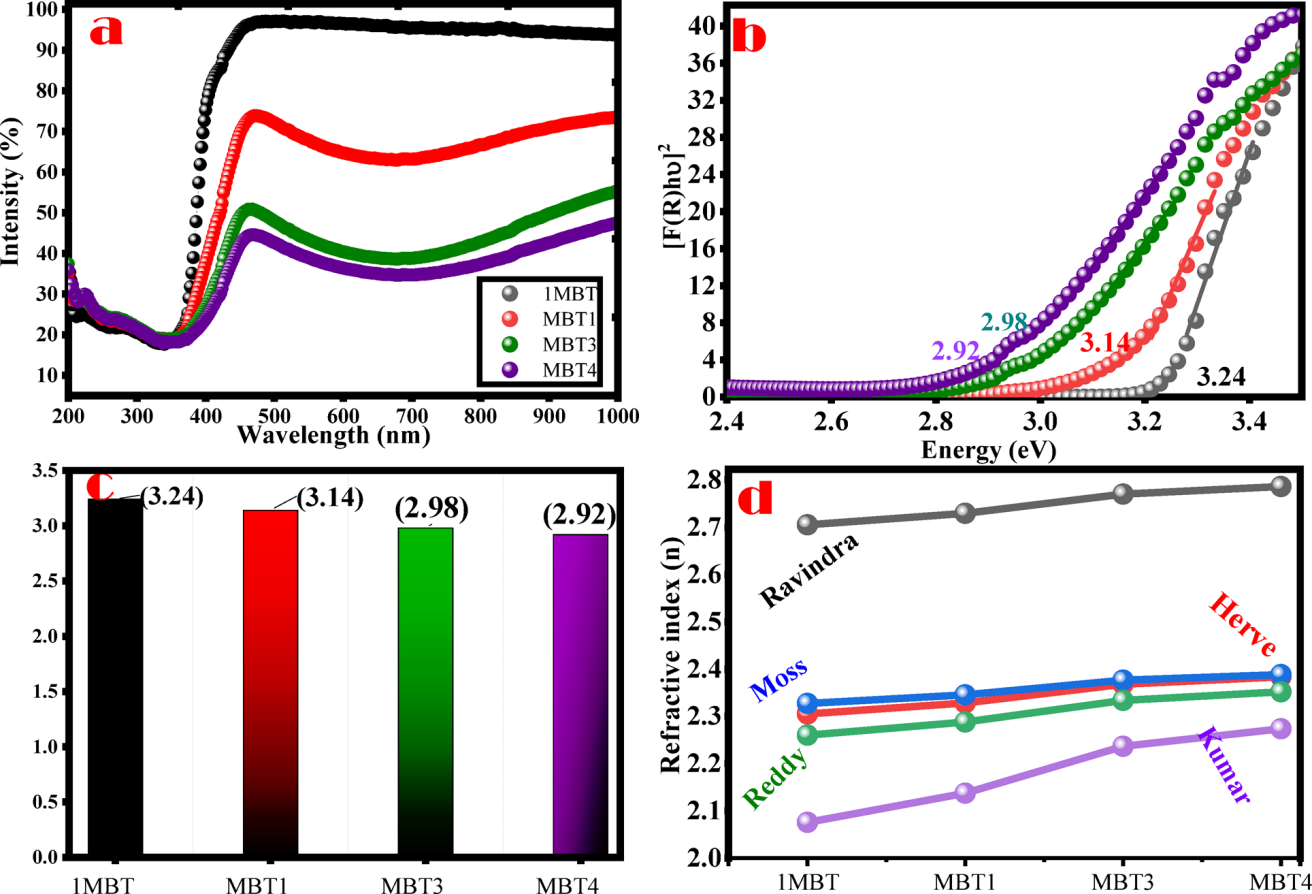
(**a**): diffuse reflectance, (**b**): Kubelka-Munk plots for optical band gap energy determination and (**c**): band gap energy values of pure and Mo doped BaTiO_3_ samples.

The optical reflectance of materials provides essential insights into their interaction with light, including their ability to reflect or absorb incident radiation across a wide wavelength range. The reflectance of BaTiO_3_ (BT) and molybdenum-doped BaTiO_3_ demonstrates a distinct pattern that highlights the impact of doping on light utilization the pure BT exhibited significantly higher reflectance compared to its doped counterparts across the measured wavelength range of 200–1000 nm (Fig. 7). This high reflectance indicates that BT reflects most incident light, particularly in the visible to near-infrared region (450–1000 nm). The higher reflectance peaked at around 95%, in this range indicating low light absorption and substantial reflection. Such behavior aligns with the intrinsic properties of BaTiO_3_ as a wide-bandgap material, which limits its ability to absorb visible light efficiently.

This high reflectance translates to limited optical absorption, restricting BT’s potential for applications such as photocatalysis under visible-light illumination. With the introduction of molybdenum as a dopant, a systematic decline in reflectance was observed as the doping concentration increased. The reflectance reduction is particularly pronounced in the visible to near-infrared range (400–1000 nm), where higher doping levels enable better absorption of light. Increasing Mo doping content led to gradual decrease in the reflectance percentage; the lowest reflectance was recorded for the highest Mo doping level (4%), where reflectance dropped significantly below that of pure BT below with reflectance percentage below 45% in the visible and near-infrared range (400–1000 nm). This significant reduction in reflectance reflects enhanced light absorption capabilities and indicates that the doping process effectively suppresses the reflective nature of BaTiO_3_ and enhances its ability to absorb visible and near-infrared light, which is critical for light-driven applications. The decreasing reflectance with increasing Mo concentration suggests that the dopant atoms create intermediate energy states within the material’s electronic structure, facilitating absorption of lower-energy photons. BT exhibits strong absorption in the ultraviolet (UV) region, with a peak around 380 nm. This absorption is linked to the electronic transitions corresponding to its intrinsic bandgap energy. However, beyond the UV range, absorption declines sharply, indicating that pure BT has minimal interaction with visible light. This limited visible-light absorption correlates with its high reflectance in this region, reaffirming that pure BT is predominantly active under UV light. The introduction of molybdenum as a dopant caused a redshift in the absorption edge. This means that the absorption band extended to longer wavelengths, progressively moving into the visible spectrum as the Mo doping concentration increased. The redshift reflects a reduction in the energy required for electronic transitions, enabling the material to absorb photons of lower energy (i.e., visible light). As the doping concentration increased, more shifts to lower energy were observed with the highest shift recorded for Mo-doped sample (4%) demonstrating the best visible-light absorption.

The optical band gap (Eg​) of pure BaTiO_3_ (BT) and Mo-doped BaTiO_3_ was determined using the Kubelka-Munk equation in conjunction with the Tauc plot method Fig. 7b. To determine the direct band gap, the linear portion of the plot was extrapolated to the point where [F(R).hν]²= 0, with F(R) representing the Kubelka-Munk Eqs.^39,40^. These techniques enable precise estimation of the band gap from diffuse reflectance data, which is crucial for understanding how Mo doping alters the electronic structure of the material. The band gap of pure BT was found to be 3.24 eV, a typical value for wide-bandgap semiconductors. This high band gap corresponds to the material’s ability to absorb ultraviolet light but limits its interaction with visible light, as photons with energy lower than 3.24 eV cannot excite electrons from the valence band to the conduction band. It is interestingly observed that Mo doping systematically reduced the band gap of BT from 3.24 eV (pure) to 2.92 eV (4% Mo) (Table 2). This gradual decrease is attributed to the incorporation of Mo ions into the BaTiO_3_ lattice, which creates intermediate energy levels within the band structure. These levels reduce the energy required for electronic transitions, enabling the material to absorb photons of lower energy. The band gap decreased in BTM1 to 3.14 eV, marking a slight shift toward visible light absorption. With increasing the Mo doping percentages, the band gap was narrowed further to 2.98 for 3% doping percentage. The lowest band gap of 2.92 eV was observed in the BTM4 sample, showing the most substantial shift toward visible light absorption among the doped samples. This trend aligns with the expected behavior wherein increased Mo incorporation introduces impurity states and promotes oxygen vacancy formation, effectively narrowing the band gap. The narrowing of the band gap with increasing Mo concentration is indicative of enhanced interaction between Mo dopants and the BaTiO_3_ lattice. Each increment in Mo concentration contributes to the introduction of additional intermediate states, thereby altering the material’s electronic properties. It is noteworthy that in a prior study, a 2% Mo-doped BaTiO_3_ sample synthesized via the solid-state route exhibited a band gap of 2.92 eV^8^. In the present work, however, the 3% Mo-doped sample shows a slightly higher band gap of 2.98 eV. This trend is justifiable when considering structural differences between the two compositions. Specifically, the XRD analysis in the current study revealed a clear phase transition from tetragonal to cubic structure occurring between 2% and 3% Mo doping. This structural change was not observed in the earlier reported 2% doped sample, which retained the tetragonal symmetry. Therefore, the divergence in band gap trends between the two studies can be attributed to the distinct crystallographic phases and defect configurations, despite the use of the same solid-state synthesis method. The reduction in band gap from 3.24 eV for pure BaTiO_3_ to 2.92 eV for 4% Mo-doped BaTiO_3_ highlights the tunability of this material’s optical properties (Fig. 7c). By altering the band structure, Mo doping enhances the material’s potential for applications requiring absorption of lower-energy photons, such as in visible-light-driven processes.

The refractive index (n) is a critical optical property that reflects how light propagates through a material. It provides insights into the interaction between light and the material’s electronic structure. For BaTiO_3_ (BT) and molybdenum (Mo)-doped BaTiO_3_, the refractive index values were calculated using five different theoretical models: Reddy, Kumar, Moss, Herve-Vandamme, and Ravindra^41–43^. These models were utilized to estimate the refractive index of the synthesized compositions.

n = [(95/Eg)]^1/4^ (Moss).

n = √ [1+ (13.6/Eg + 3.47)^2^] (Hervé and Vandamme).

*n* = 4.084–0.62Eg (Ravindra et al.)

*n* = 3.3668Eg^− 0.32234^ (Kumar and Singh).

n=(154/Eg-0.365)^1/4^ (Reddy and Anjayenulu).

These models were selected because they are widely reported in the literature and emphasize different empirical correlations between *n* and *E*_*g*_. The Reddy and Kumar models are refined forms applicable to oxide materials, while Moss is frequently used for semiconductors with relatively large band gaps, whereas the Herve–Vandamme relation accounts for ionic/covalent bonding effects. Across all models, the refractive index increases systematically as Mo concentration rises from 1% to 4% (Fig. 7d). This trend is consistent with the band gap reduction observed in Mo-doped BaTiO_3_, as refractive index (n) is inversely related to the optical band gap (Eg​). The increase in n with Mo doping suggests enhanced interaction between light and the material due to changes in its electronic polarizability. This can be attributed to the introduction of lattice defects caused by the incorporation of Mo ions into the BaTiO_3_ structure^44,45^.

$$\:{Mo}_{2}{O}_{3}\underrightarrow{{BaTiO}_{3}}\:{Mo}_{Ti}^{\bullet\:\:}+{V}_{Ti}^{{\prime\:}{\prime\:}{\prime\:}{\prime\:}}+3{O}_{o}^{\times\:}$$

The refractive index values (Table 2) indicate improved optical confinement in the doped materials, which can influence their performance in optical and photonic applications. The reduction in the band gap from 3.24 eV for pure BaTiO_3_ to 2.92 eV for 4% Mo-doped BaTiO_3_ correlates with a systematic increase in refractive index across all models. The Reddy model provides refractive index values (≈ 2.7–2.8) that are closest to experimental reports for BaTiO_3_, suggesting it is the most suitable among the tested relations. The other models, though less accurate, are included here for completeness and to illustrate the spread in predicted values depending on the chosen correlation. This relationship is consistent across the theoretical models, highlighting the reliability of the calculations and the impact of Mo doping on the optical properties of BaTiO_3_. This ability to control the refractive index through doping allows for precise tuning of optical properties of BaTiO_3_, making it suitable for applications like waveguides, sensors, and dielectric mirrors.

**Table 2:** Band gap and refractive index (n) values of pure and mo doped BaTiO_3_ samples compositions.

Samples	Band gap (eV)	Reddy	Kumar	Moss		Herve-vandamme	Ravindra
BT	3.24	2.70533	2.30488	2.32699		2.26009	2.0752
BTM1	3.14	2.72938	2.32829	2.3453		2.28763	2.1372
BTM3	2.98	2.76757	2.36532	2.37418		2.33069	2.2302
BTM4	2.92	2.79181	2.38873	2.39239		2.3576	2.286

### Dielectric and ac electrical properties

**Fig. 8:**
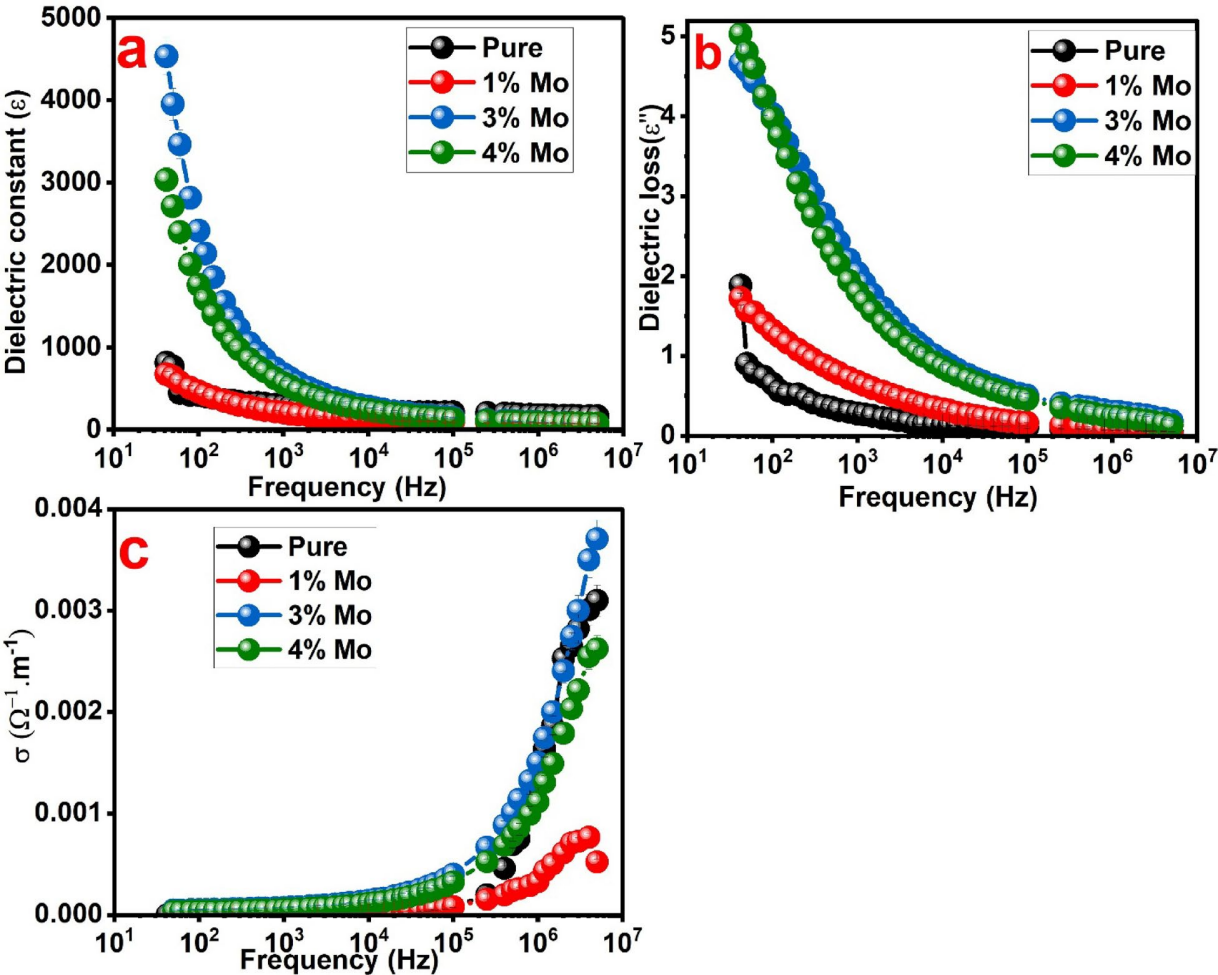
(**a**): dependence of dielectric constant on frequency, (**b**): dependence of dielectric loss tangent (δ) on frequency and (**c**): dependence of ac electrical conductivity (σ) on frequency of BaTiO_3_, BaTi_0.99_Mo_0.01_O_3_, BaTi_0.97_Mo_0.03_O_3_ and BaTi_0.96_Mo_0.04_O_3_ samples.

Figure 8a displays the correlation between room temperature dielectric constant and frequency of BaTiO_3_, BaTi_0.99_Mo_0.01_O_3_, BaTi_0.97_Mo_0.03_O_3_ and BaTi_0.96_Mo_0.04_O_3_ samples. As can be seen, the dielectric constant performance of all samples was affected by changing the frequency and it is clearly decreases with increasing the applied frequency. The incorporation of 3 and 4% Mo ions greatly improves the dielectric constant values particularly at low frequency. For example, at 100 Hz the measured dielectric constant of BaTiO_3_, BaTi_0.99_Mo_0.01_O_3_, BaTi_0.97_Mo_0.03_O_3_ and BaTi_0.96_Mo_0.04_O_3_ samples was estimated to be 420, 462, 2416 and 1758, respectively. By comparing between samples, the highest dielectric constant value was achieved for BaTi_0.97_Mo_0.03_O_3_ composition. The remarkable dielectric constant of this composition is promising for energy storage systems, especially at low frequency. However, further increasing the Mo content to 4% (BaTi₀.₉₆Mo₀.₀₄O₃) led to enhanced particle agglomeration, as confirmed by SEM analysis. Such agglomeration can cause localized charge buildup and uneven polarization, which compromise dielectric uniformity and stability, ultimately leading to a reduction in the dielectric constant at excessive doping levels. The manner of the dielectric constant of BaTiO_3_, BaTi_0.99_Mo_0.01_O_3_, BaTi_0.97_Mo_0.03_O_3_ and BaTi_0.96_Mo_0.04_O_3_ samples is in accordance with the relaxation mechanism and polarization route observed in numerous semiconducting perovskite and oxide compositions. In comparison to an earlier study for Mo, V and Co, the dielectric constant at 42 Hz revealed dielectric constants of 14,263, 743, and 1658 for BaTi_0.99_Mo_0.01_O_3_, BaTi_0.97_Mo_0.03_O_3_ and BaTi_0.96_Mo_0.04_O_3_, respectively. Although V doping at 2% produced a colossal dielectric constant, Mo doping consistently demonstrated substantial enhancement relative to Co doping, and in the present study, the optimized 3% Mo-doped composition far exceeded the dielectric performance of the 2% Mo-doped sample reported earlier^8^. As reported in literature, the detected dielectric constant behavior of the manufactured compositions can be clarified established on the well-known model of Maxwell–Wagner^46,47^. In agreement with this model, the BaTiO_3_, BaTi_0.99_Mo_0.01_O_3_, BaTi_0.97_Mo_0.03_O_3_ and BaTi_0.96_Mo_0.04_O_3_ compositions are proposed to be consists of grain boundaries have feeble conducting which separate the grains which have high conducting. When the electric field is applied, the charge carriers (electrons) are collected at the grain boundaries which possess poor conductivity. The high dielectric constant is produced at low frequency owing to the large polarizability coming from the accumulation of the charge carriers at the grain boundaries^46,47^. While at greater frequencies the electric dipoles of BaTiO_3_, BaTi_0.99_Mo_0.01_O_3_, BaTi_0.97_Mo_0.03_O_3_ and BaTi_0.96_Mo_0.04_O_3_ compositions cannot track the changes in the electric field; therefore, the growth of frequency reduces the dielectric constant of these samples. In short words, the dielectric constant at lower frequency of BaTiO_3_, BaTi_0.99_Mo_0.01_O_3_, BaTi_0.97_Mo_0.03_O_3_ and BaTi_0.96_Mo_0.04_O_3_ compositions are expected to come from many sorts of polarization like electronic polarization, interfacial polarization, dipolar polarization, electronic polarization and ionic polarization besides the effect of oxygen vacancies and defects^46,47^.

Figure 8b represents the relation between dielectric loss tangent (δ) and frequency of BaTiO_3_, BaTi_0.99_Mo_0.01_O_3_, BaTi_0.97_Mo_0.03_O_3_ and BaTi_0.96_Mo_0.04_O_3_ compositions at room temperature. At all frequencies, BaTi_0.97_Mo_0.03_O_3_ and BaTi_0.96_Mo_0.04_O_3_ compositions reveals high δ values compared to BaTiO_3_ and BaTi_0.99_Mo_0.01_O_3_ samples. Similar to dielectric constant, the δ performance was reduced with growing the frequency. At low frequency, the high values of dielectric loss tangent can be related to defects, moisture and impurities in BaTiO_3_, BaTi_0.99_Mo_0.01_O_3_, BaTi_0.97_Mo_0.03_O_3_ and BaTi_0.96_Mo_0.04_O_3_ compositions^48^. Figure 8c gives the relation between ac electrical conductivity (σ, Ω^−1^cm^−1^) and frequency of BaTiO_3_, BaTi_0.99_Mo_0.01_O_3_, BaTi_0.97_Mo_0.03_O_3_ and BaTi_0.96_Mo_0.04_O_3_ compositions at room temperature. It can be noticed that the electrical conductivity was greatly increased at high frequency while at low frequency the changes of the electrical conductivity are small. BaTi_0.97_Mo_0.03_O_3_ sample exhibits the highest ac electrical conductivity. The ac electrical conductivity performance of BaTiO_3_, BaTi_0.99_Mo_0.01_O_3_, BaTi_0.97_Mo_0.03_O_3_ and BaTi_0.96_Mo_0.04_O_3_ compositions at small frequency-independent and large frequency-dependent representing the relaxation manner of the ac electrical conductivity. The steady growth of the ac electrical conductivity of BaTiO_3_, BaTi_0.99_Mo_0.01_O_3_, BaTi_0.97_Mo_0.03_O_3_ and BaTi_0.96_Mo_0.04_O_3_ compositions with growing the applied frequency can be clarified by using the famous interfacial Maxwell-Wagner model^48,49^. At the grain boundaries of these samples a potential barrier is produced which remarkably effect on the transfer of the charge carriers which as like particles in a box^48,49^. The weak increases of electrical conductivity at low frequency of BaTiO_3_, BaTi_0.99_Mo_0.01_O_3_, BaTi_0.97_Mo_0.03_O_3_ and BaTi_0.96_Mo_0.04_O_3_ compositions can be attributed to the small number of the charge carriers which can tunnel over the formed potential barrier, leading to feeble ac electrical conductivity values. After rising the applied frequency, the charge carriers have the appropriate energies to transfer over the formed potential barrier, leading to high ac electrical conductivity values^48,49^.

### Photodegradation study

**Fig. 9:**
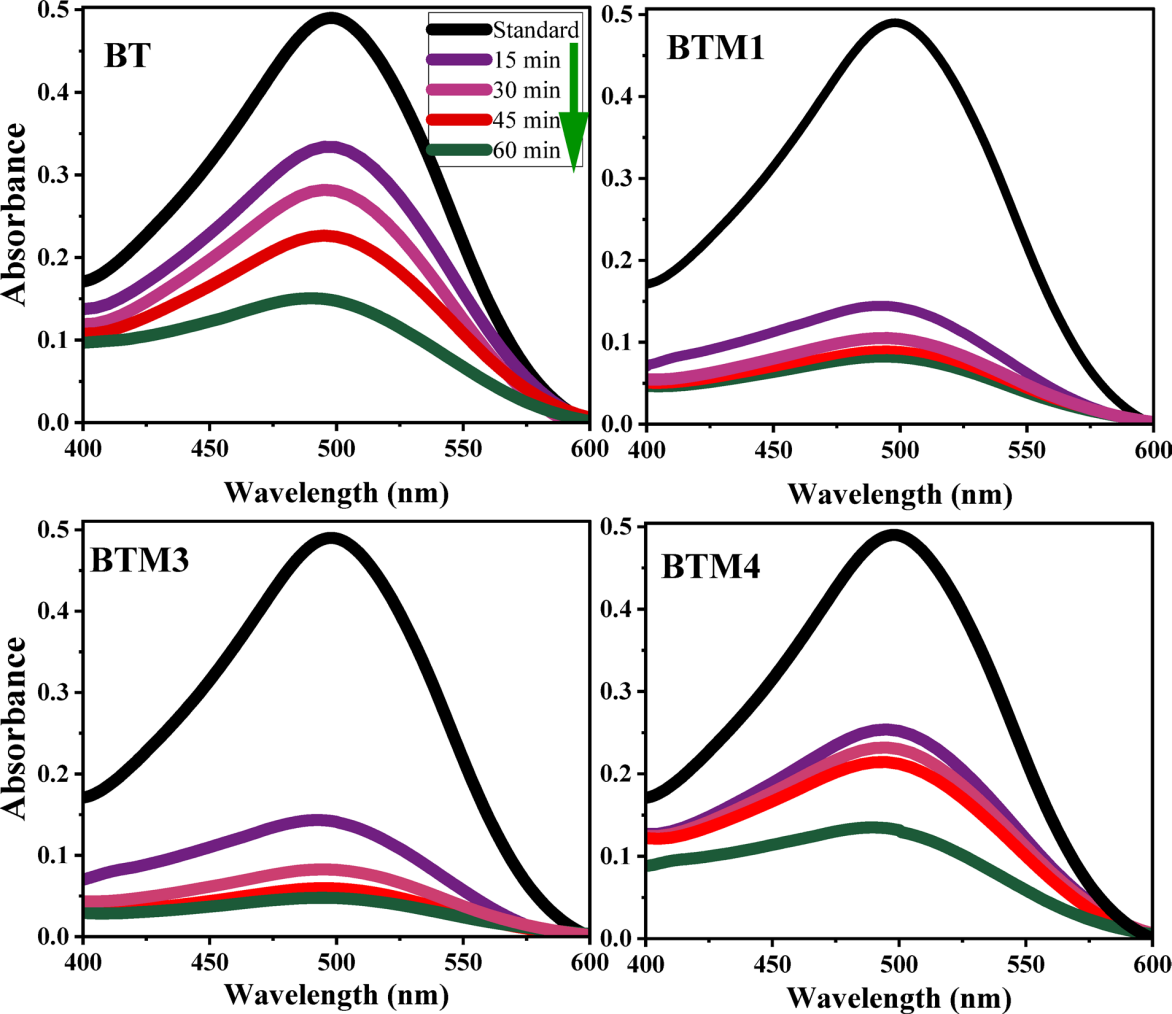
UV–Visible absorbance of Congo red solution (10 ppm) in presence of pure and Mo doped BaTiO_3_ samples.

The degradation of Congo Red (CR) dye was monitored over 60 min, with absorbance measured at regular intervals (Fig. 9). A significant decline in the absorption peak at the dye’s characteristic wavelength indicates progressive breakdown of the chromophore structure, leading to mineralization. The trends in absorption reflect the photocatalytic activity of BaTiO_3_ and its molybdenum-doped variants. The pure BaTiO_3_ (BT) sample demonstrated a photocatalytic degradation efficiency of 69.5% over 60 min. The wide bandgap (3.24 eV) of the pure BT sample restricts its visible-light absorption; the moderate performance arises from its limited ability to harness visible-light photons, alongside possible recombination of photogenerated charge carriers. Doping BaTiO_3_ with molybdenum (Mo) at concentrations (1, 3 and 4%) significantly alters the material’s photocatalytic behavior. The introduction of Mo narrows the bandgap, enhancing visible-light absorption and improving photocatalytic performance up to an optimal doping level. The slight narrowing of the bandgap in BTM1 (3.14 eV) improves visible-light absorption, leading to enhanced degradation efficiency (85.7%) compared to pure BT sample. This suggests that a small amount of Mo effectively enhances light absorption and reduces charge-carrier recombination. The narrowed bandgap allows maximum visible-light utilization, and the Mo doping effectively reduces electron-hole recombination, resulting in high degradation efficiency of the dye within 60 min. Slight increase in the Mo doping to 3% led to an optimal doping level that significantly enhances photocatalytic activity. The further decline in the bandgap (2.99 eV) allows maximum visible-light utilization, resulting in 90% degradation of the dye within 60 min. This sample exhibits the best photocatalytic performance. Further increase in the Mo doping percentage (4%,) does not improve degradation efficiency, nevertheless it narrows the bandgap. Instead, the excess dopant likely introduces recombination centers, hindering photocatalytic performance. Both BTM3 and BTM4 show performance comparable to pure BT, indicating a decline from the optimal doping level. The results highlight that Mo doping enhances BaTiO_3_’s photocatalytic activity by reducing the bandgap and improving visible-light absorption. However, doping beyond the optimal concentration (3%) leads to diminished returns due to recombination effects. The findings emphasize the delicate balance between enhancing light absorption and maintaining efficient charge separation.

**Fig. 10:**
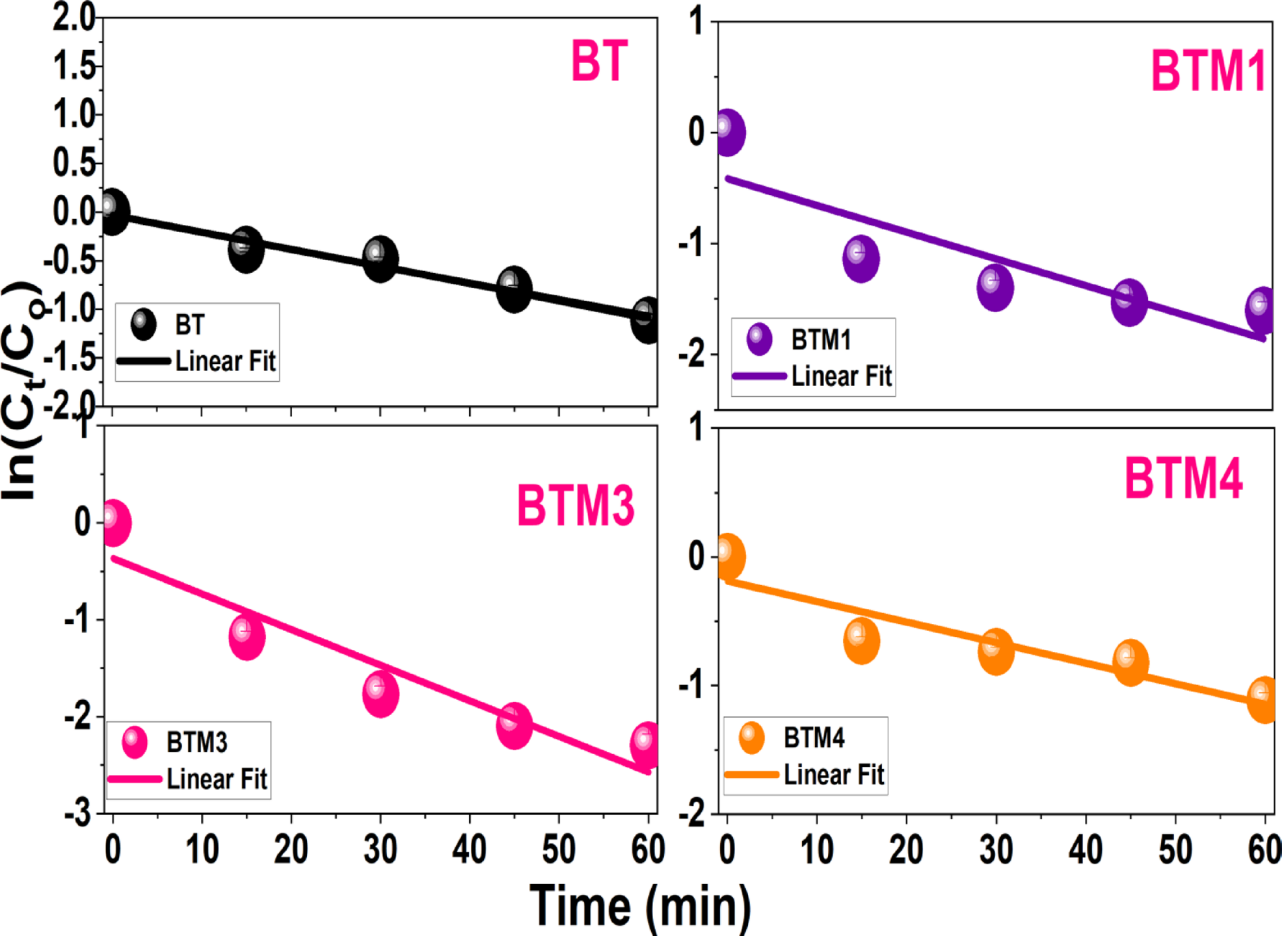
Kinetic profile, expressed as the natural logarithm of the concentration ratio (Ct/C0) over time for degradation of Congo red using pure and Mo doped BaTiO_3_ samples.

The photocatalytic degradation kinetics of Congo Red dye over BaTiO_3_ (BT) and Mo-doped BaTiO_3_ samples (BTM1, BTM3, BTM4) were analyzed using pseudo-first-order kinetics, as indicated by the linear fitting of ln(C_t_/C_0_) versus time plots (Fig. 10). The corresponding rate constants were calculated to be 0.0175, 0.0240, 0.0367, and 0.0178 min^− 1^ for BT, BTM1, BTM3, and BTM4, respectively. These values reflect a clear enhancement in degradation rate upon Mo doping, with the BTM3 sample exhibiting the highest rate constant, suggesting remarkable photocatalytic performance. This improvement is likely due to an optimal Mo concentration that promotes better charge separation and reduces electron-hole recombination, enhancing the efficiency of photocatalytic reactions. In contrast, while BTM1 also shows improved activity over the pure BT, the enhancement is moderate. The performance of BTM4, however, is nearly equivalent to that of undoped BT, indicating that excessive Mo incorporation may lead to increased defect density or structural disorder, which negatively impacts photocatalytic efficiency. Furthermore, the higher Mo doping levels (4%) lead to increased particle agglomeration, as observed in SEM micrographs. Such agglomeration can reduce the effective surface area available for photocatalytic reactions, thereby limiting the number of active sites for dye adsorption and degradation. Therefore, the data affirm that controlled Mo doping plays a critical role in tuning the photocatalytic activity of BaTiO_3_-based materials. Compared with V- and Co-doped BaTiO_3_ compositions reported in a previous study, Mo-doped BaTiO_3_ demonstrates markedly superior photocatalytic performance, achieving Congo red (10 ppm) degradation efficiencies of 100% and 90% for 2 and 3% Mo doping, respectively, within 60 min under sunlight. In contrast, V- and Co-doped samples attained lower degradation efficiencies of 80% and 76%, respectively, under similar conditions. This enhanced activity is linked to the multivalent nature of Mo, which induces a higher density of oxygen vacancies, improved crystallinity, and more effective charge separation, as well as slightly greater band gap reduction (2.92 eV) compared to V (2.96 eV) and Co (2.98 eV) doping. These combined effects underscore Mo’s distinctive advantage in optimizing both dielectric and photocatalytic responses in BaTiO_3_ systems.

The enhanced photocatalytic activity of Mo-doped BaTiO_3_ samples for Congo red (CR) degradation under sunlight irradiation can be attributed to several synergistic effects, including improved visible light absorption, increased surface oxygen vacancies, and effective charge carrier separation induced by Mo doping. Upon exposure to sunlight, Mo-doped BaTiO_3_ absorbs photons with energy equal to or greater than its band gap, leading to the excitation of electrons (e^−^) from the valence band (VB) to the conduction band (CB), thereby generating holes (h^+^) in the VB^50,51^:

BaTi_1 − x_Mo_x_O_3_ + hν → e_CB−_ + h_VB+_.

The photogenerated electrons and holes migrate to the surface of the photocatalyst, where they initiate redox reactions. The electrons in the CB reduce dissolved oxygen molecules to form superoxide radicals (^⋅^O_2_^−^​):

e^−^ + O_2_→^⋅^O_2_^−^

Simultaneously, the holes in the VB can oxidize water molecules or hydroxide ions adsorbed on the catalyst surface to produce hydroxyl radicals (⋅OH):

h^+^ + H_2_O → ^⋅^OH + H^+^.

or.

h^+^ + OH^−^ → ^⋅^OH.

Both superoxide (^⋅^O_2_^−^and hydroxyl (^⋅^OH) radicals are highly reactive oxidative species that attack and break down the CR dye molecules into smaller, less toxic intermediates, ultimately leading to complete mineralization into CO₂, H₂O, and other innocuous end-products:

^⋅^O_2_^−^, ^⋅^OH + Congo red→ Degraded products. The high photocatalytic performance of the 3% Mo-doped sample (BTM3), which achieved about 90% degradation of Congo red within 60 min, can be primarily linked to band gap narrowing (from 3.14 eV in pure BT to 2.99 eV in BTM3), enhancing sunlight harvesting, efficient charge carrier separation, reducing recombination rates and the formation of multivalent Mo species (Mo^3+^/Mo^4+^/Mo^6+^), which may serve as electron traps to suppress recombination Mo^n+^ + eˉ → Mo^(n+)−1^ (delay or hinder the recombination of h^+^ and eˉ) Mo^n+^ + O_2_ → Mo^(n+)+1^ + O_2_˙ˉ. In addition to reduced electron–hole recombination and the formation of intermediate states due to Mo incorporation, oxygen vacancies and related defect states could also play a pivotal role in improving visible-light photocatalytic performance. These defects, introduced through lattice distortion and charge compensation during Mo substitution, act as shallow donor levels that enhance charge carrier separation and extend light absorption into the visible range. This synergistic effect between Mo-induced states and oxygen vacancy levels facilitates more efficient generation and transfer of reactive species, thereby accelerating the degradation process. The combined effects of Mo doping and surface band bending significantly participated in enhanced photocatalytic activity of Mo–BaTiO_3_. Mo incorporation introduces intermediate energy levels within the band gap, leading to band gap narrowing and improved visible-light harvesting. At the same time, Mo doping promotes surface defects that serve as electron or hole trapping sites. Under visible-light irradiation, upward band bending at the BaTiO_3_ surface establishes an internal electric field that drives photogenerated electrons into the bulk and holes toward the surface. This spatial separation, together with Mo-induced defect trapping, reduces recombination and promotes the formation of •O₂⁻ and •OH radicals, which are the key species responsible for dye degradation. Mo incorporation introduces intermediate energy levels within the band gap, leading to band gap narrowing and improved visible-light harvesting. At the same time, Mo doping promotes surface defects that serve as electron or hole trapping sites. Under visible-light irradiation, upward band bending at the BaTiO_3_ surface establishes an internal electric field that drives photogenerated electrons into the bulk and holes toward the surface. This spatial separation, together with Mo-induced defect trapping, reduces recombination and promotes the formation of •O₂⁻ and •OH radicals, which are the key species responsible for dye degradation. This multistep mechanism highlights the importance of Mo incorporation in tailoring the structural, optical, and electronic properties of BaTiO_3_ for enhanced solar-driven environmental remediation.

**Fig. 11:**
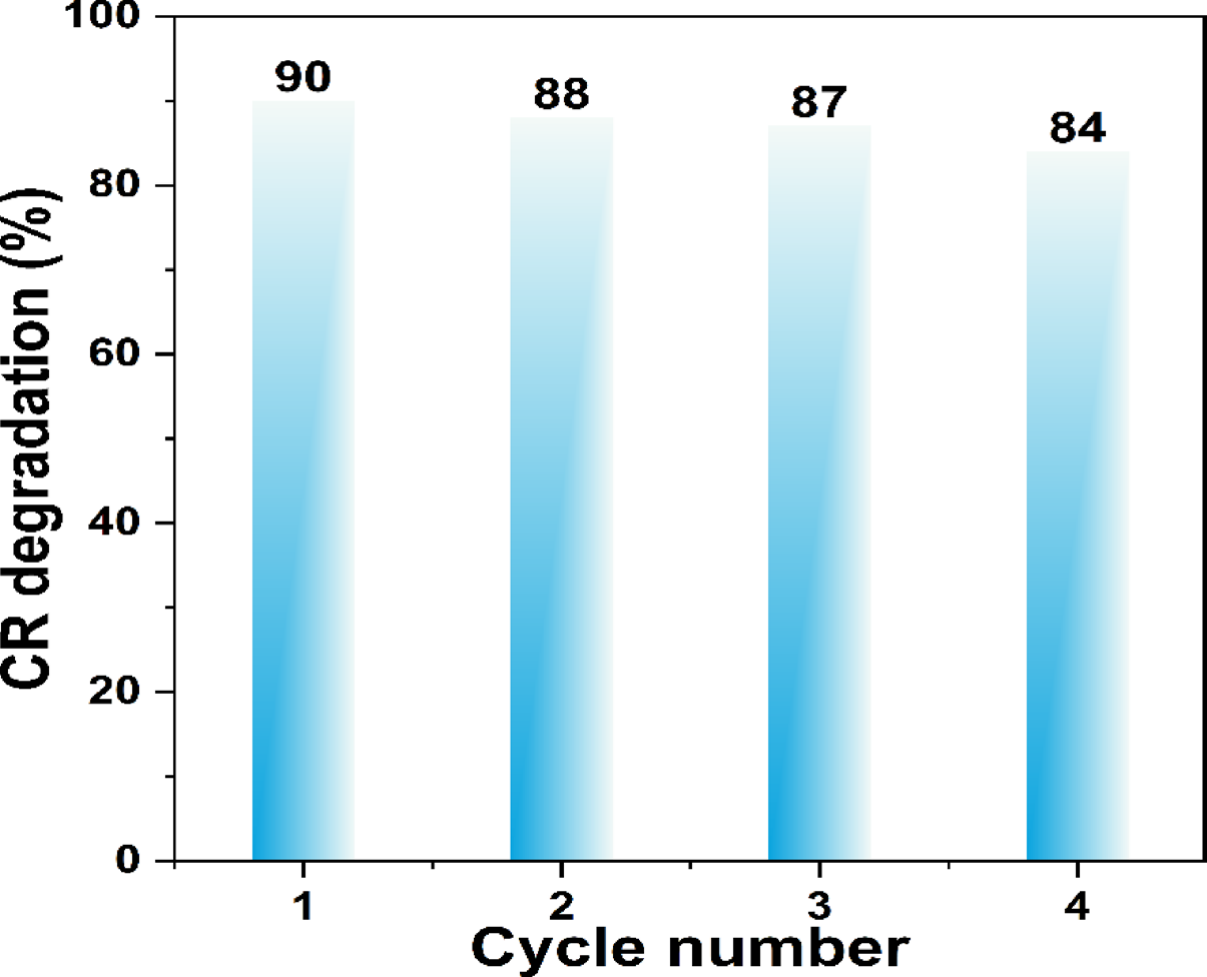
Recovery cycles of BaTi_0.97_Mo_0.03_O_3_ against 10 ppm Congo red at 60 min.

To assess the durability of the Mo-doped BaTiO_3_ photocatalysts, cycling experiments were performed under identical visible-light irradiation conditions for four consecutive runs. The recyclability of the BaTi_0.97_Mo_0.03_O_3_ photocatalyst was evaluated through consecutive recovery tests. After each cycle, the photocatalyst was carefully washed with 30 mL of doubly distilled water, followed by drying at 120 °C to remove any adsorbed residues before reuse. The catalyst exhibited an initial degradation efficiency of 90%, which slightly decreased to 88%, 87%, and 85% over four successive cycles (Fig. 11). This minor reduction can be ascribed to inevitable handling losses during washing and drying. Overall, the results confirm that the BaTi_0.97_Mo_0.03_O_3_ catalyst maintains good stability and reusability, highlighting its potential as an efficient and recyclable photocatalyst for practical applications.

In the context of the present study, Mo-doped BaTiO_3_ catalysts demonstrated high photocatalytic efficiency toward Congo red degradation under sunlight, highlighting their potential for environmental remediation. This approach is inherently more scalable than solution-based methods, making it attractive for potential industrial applications. However, for large-scale wastewater treatment, further optimization will be needed to facilitate catalyst recovery, such as immobilization on suitable supports or magnetic modification, as well as testing under complex wastewater conditions. These aspects are beyond the scope of the current work and could be addressed in future studies.

## Conclusion

Pure and Mo-doped BaTiO_3_ (BTO) powders were successfully synthesized via a solid-state reaction route. X-ray diffraction confirmed the tetragonal perovskite phase in all samples, with observable peak shifts toward lower 2θ values upon Mo doping, indicative of lattice expansion due to the substitution of smaller Ti^4+^ ions (0.605 Å) with larger Mo^3+^/Mo^4+^ ions (0.69 Å and 0.65 Å, respectively). XPS revealed the coexistence of Mo^3+^, Mo^4+^, and Mo^6+^ oxidation states, and showed a clear presence of Ti^3+^ states alongside Ti^4+^, indicating the generation of oxygen vacancies for charge compensation. SEM images revealed relatively uniform grain morphology, while EDX analysis verified the elemental composition without any unexpected impurities. Optical studies showed that the band gap energy of pure BTO decreased from 3.24 eV to 2.91 eV for the 4 wt% Mo-doped sample, as calculated using the Kubelka-Munk function and Tauc plots. This red shift improves visible light absorption and is reflected in the increase of refractive index values supporting stronger light–matter interaction. Dielectric characterization demonstrated enhanced room-temperature relative permittivity (εʹ) and lower dielectric loss (tan δ) with increasing Mo content, confirming the impact of Mo incorporation on polarization behavior and energy storage capability. Photocatalytic tests against Congo red dye under showed that the degradation rate constants improved from 0.01754 min⁻¹ (pure BTO) to 0.03673 min⁻¹ (MBT3), signifying more than a two-fold enhancement in performance under solar illumination. Altogether, the study confirms that Mo doping is an effective strategy to tune the optical and dielectric properties of BaTiO_3_, making it a promising multifunctional material for photocatalytic and electronic applications. The recovery tests confirmed that BaTi_0.97_Mo_0.03_O_3_ retains high activity (90–85% over repeated cycles), underscoring its practical potential for environmental applications under visible light. These findings present incremental yet meaningful advances that enrich the current understanding of perovskite-based photocatalysts and provide a foundation for further optimization in industrial-scale water treatment and highlight the potential of tailored Mo-doping into BaTiO_3_ strategy to optimize photocatalytic materials for environmental remediation.

## Data Availability

Data will be made available on request. Please contact Mohammed Wahba at mohamedwahba12@gmail.com.
